# A Case of Drug‐Induced Pancreatitis

**DOI:** 10.1002/ccr3.71915

**Published:** 2026-01-21

**Authors:** Muskan Shrestha, Pratick Shrestha, Adarsha Mahaseth, Abhishek Shrestha, Prabin Duwadee, Rajat K. Shah

**Affiliations:** ^1^ Manmohan Cardiothoracic Vascular and Transplant Center Kathmandu Nepal; ^2^ Vayodha Hospitals Kathmandu Nepal; ^3^ Nepalese Army Institute of Health Sciences (NAIHS) Kathmandu Nepal; ^4^ Nepal APF Hospital Kathmandu Nepal; ^5^ Dhading District Hospital Dhading Nepal; ^6^ International Friendship Children Hospital Kathmandu Nepal

**Keywords:** acute pancreatitis, adverse drug reaction, case report, ciprofloxacin, drug‐induced pancreatitis, fluoroquinolones

## Abstract

Drug‐induced pancreatitis (DIP) is a rare but important cause of acute abdominal pain, and ciprofloxacin‐associated cases are exceptionally uncommon. We report a 35‐year‐old woman with no significant medical history who developed severe epigastric pain radiating to the back, accompanied by nausea and vomiting, 3 days after initiating self‐prescribed ciprofloxacin for diarrhea. Laboratory tests revealed markedly elevated serum amylase and lipase, and contrast‐enhanced CT confirmed acute interstitial pancreatitis. Common causes including gallstones, alcohol use, hypertriglyceridemia, hypercalcemia, and viral hepatitis were excluded. Ciprofloxacin was discontinued, and the patient was managed with intravenous fluids, bowel rest, and analgesia, leading to rapid clinical and biochemical improvement within 4 days. Given the temporal relationship, exclusion of alternative etiologies, and supportive causality assessments (Naranjo Adverse Drug Reaction Probability Scale & Badalov Classification), ciprofloxacin was considered the probable cause. This case underscores the importance of considering DIP in patients presenting with new‐onset abdominal pain during fluoroquinolone therapy, as prompt drug withdrawal and supportive care can result in swift recovery and prevent complications.

## Key Clinical Message

Acute pancreatitis may rarely occur within days of starting ciprofloxacin. Clinicians should suspect drug‐induced pancreatitis in patients with new abdominal pain during fluoroquinolone therapy, as prompt discontinuation and supportive care can lead to rapid recovery.

## 1 Introduction

Drug‐induced pancreatitis (DIP) is an inflammation of the pancreas resulting from the activation of digestive enzymes within the pancreatic tissue, caused by a medication. It is rare, accounting for a small proportion of acute pancreatitis cases, but it remains an important diagnosis to consider, especially when common causes are absent. A systematic review of case reports identified more than 200 unique medications as possible causative agents for DIP [1]. Commonly associated classes include antiretrovirals, antibiotics, antifungals, chemotherapeutics, anticonvulsants, diuretics, statins, acetaminophen, opioids, and antihypertensives [2, 3].

Ciprofloxacin, a widely prescribed broad‐spectrum fluoroquinolone, is FDA‐approved for urinary tract infections, community‐acquired pneumonia, sexually transmitted infections, prostatitis, typhoid fever, acute infectious colitis, traveler's diarrhea, and skin, bone, and joint infections. It is especially valued for activity against Gram‐negative organisms such as *Escherichia coli* and *Pseudomonas aeruginosa* [1, 4, 5]. At therapeutic doses, ciprofloxacin most frequently causes gastrointestinal side effects (nausea, diarrhea). However, FDA‐issued boxed warnings also highlight serious risks: tendinitis and tendon rupture; peripheral neuropathy; neuropsychiatric events (agitation, tremors, hallucinations, psychosis, seizures); QT prolongation; hyper‐ and hypoglycemia; photosensitivity; and an association with aortic aneurysm or dissection [5]. Despite this broad toxicity profile, pancreatitis following ciprofloxacin remains rare. Here we report a case of acute pancreatitis occurring shortly after ciprofloxacin initiation highlighting the need for vigilance and prompt recognition of this uncommon adverse effect.

## 2 Case Presentation

### 2.1 Case History and Examination

A 35‐year‐old woman with no significant past medical history presented to the emergency department with severe upper abdominal pain of 2 days' duration. The pain was sharp, occasionally radiating to the back, and was partially relieved by bending forward. It was associated with nausea and vomiting.

Five days prior, she had experienced watery, non‐bloody diarrhea with mild abdominal discomfort, for which she self‐medicated with ciprofloxacin (500 mg twice daily) obtained from a local pharmacy. The diarrheal symptoms resolved; however, on the third day of antibiotic therapy, she developed new‐onset severe epigastric pain. She denied alcohol intake, smoking, illicit drug use, abdominal trauma, recent surgery, endoscopic procedures, or chronic medication use.

On admission, she was febrile (38.5°C), with blood pressure 110/80 mmHg, pulse 100/min, and respiratory rate 20/min. On abdominal examination, there was tenderness localized to the epigastrium without rigidity, guarding, or organomegaly. Bowel sounds were normal, and the remainder of the systemic examination was unremarkable.

**FIGURE 1:**
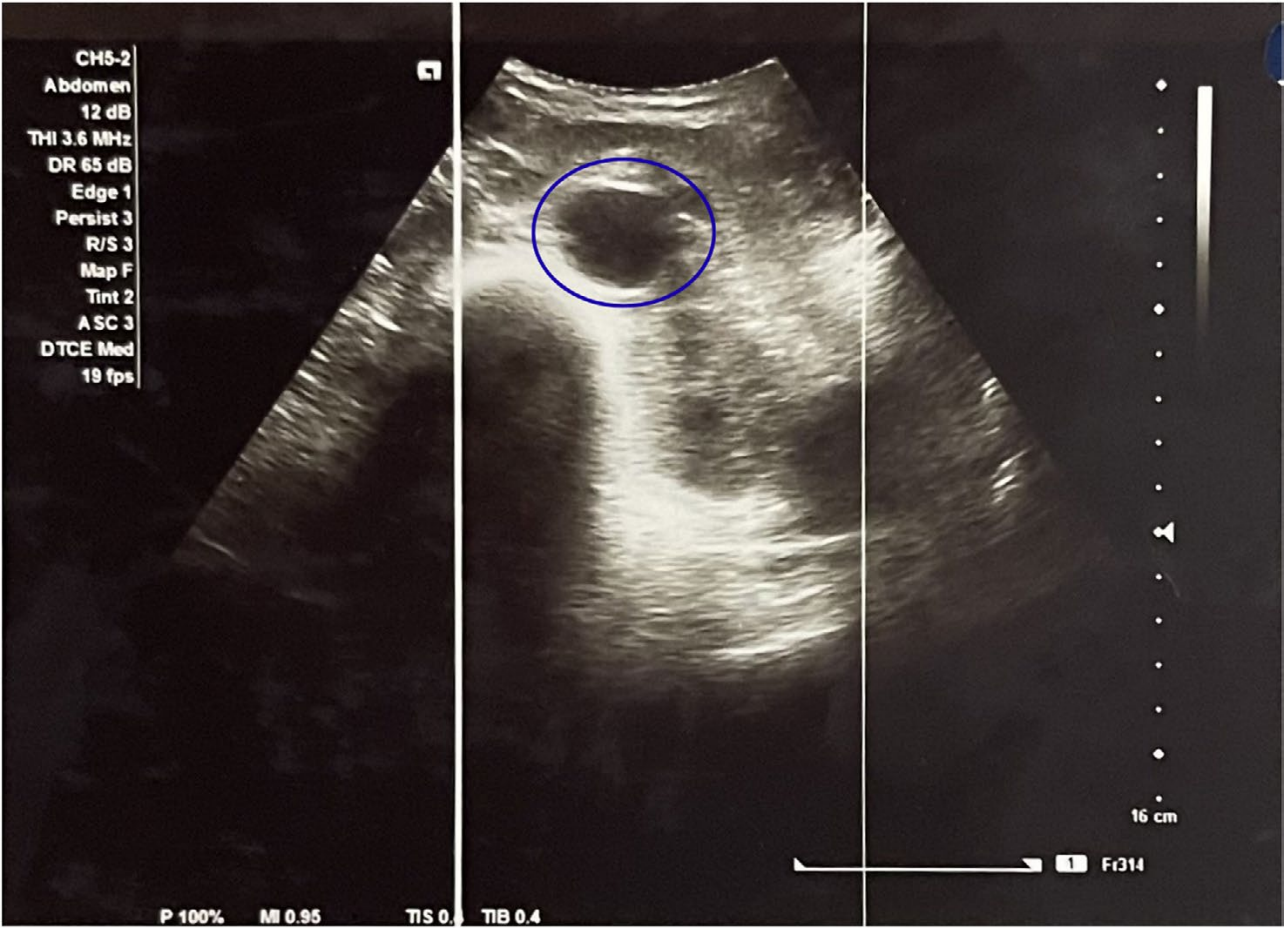
Abdominal USG showing normal well‐distended, uniformly thin‐walled gallbladder with no intraluminal stones or biliary sludge (blue circle).

**FIGURE 2:**
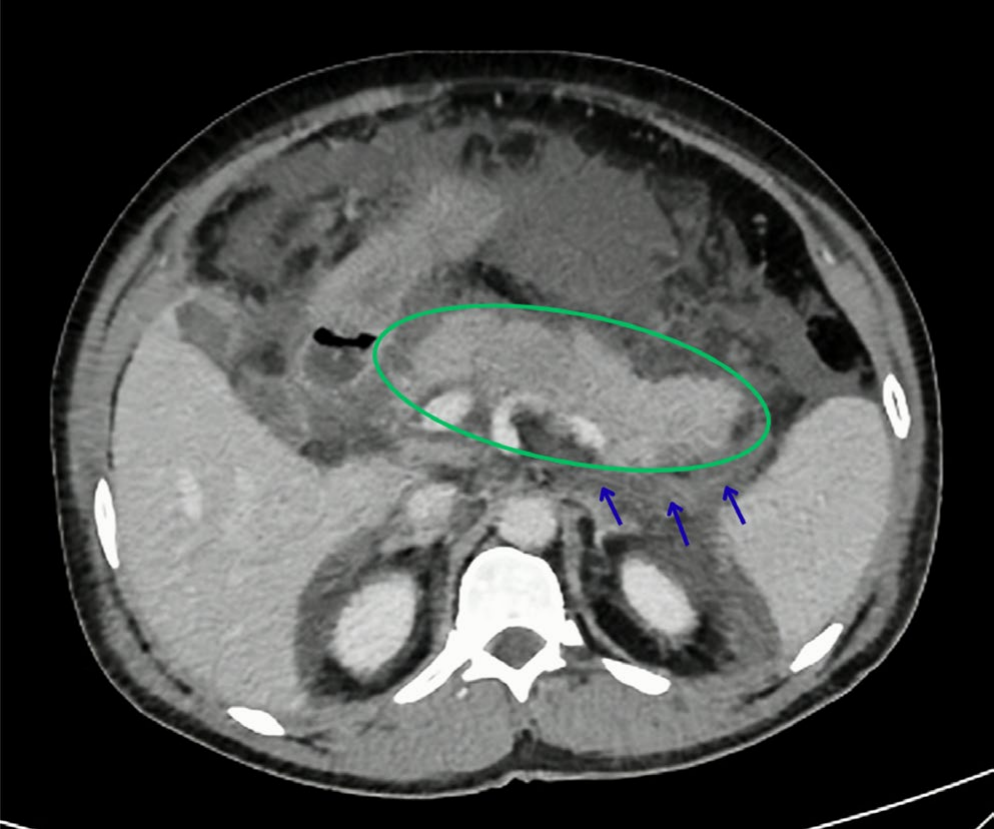
Axial CECT scan of the abdomen showing mild enlargement of the pancreas (green circle), accompanied by significant peripancreatic fat strandings (blue arrows).

Laboratory investigations revealed markedly elevated serum amylase and lipase, with mild leukocytosis and elevated CRP. Renal and liver function tests, electrolytes, calcium, lipid profile, and ethanol levels were within normal ranges (Table 1). Abdominal ultrasonography showed a normal gallbladder without stones or biliary sludge (Figure 1). A hepatitis panel was negative (Table 2). Contrast‐enhanced CT abdomen demonstrated acute interstitial pancreatitis with mild pancreatic enlargement and peripancreatic fat stranding (Figure 2).

**TABLE 1:** CBC, CRP, CMP, lipid panel, and ethanol level.

Test	Result	Reference range
WBC (white blood cells)	12 × 10^9^/L	4.0–11.0 × 10^9^/L
RBC (red blood cells)	4.9 × 10^12^/L	M: 4.7–6.1; F: 4.2–5.4 × 10^12^/L
Hemoglobin	13.8 g/dL	M: 13.5–17.5; F: 12.0–15.5 g/dL
Hematocrit	44%	M: 41%–53%; F: 36%–46%
MCV	88 fL	80–100 fL
MCH	29 pg	27–33 pg
MCHC	34 g/dL	32–36 g/dL
RDW	13.50%	11.5%–14.5%
Platelet count	220 × 10^9^/L	150–450 × 10^9^/L
Sodium	139 mmol/L	135–145 mmol/L
Potassium	4.2 mmol/L	3.5–5.0 mmol/L
Chloride	102 mmol/L	98–107 mmol/L
Bicarbonate (CO_2_)	24 mmol/L	22–29 mmol/L
BUN (blood urea nitrogen)	12 mg/dL	7–20 mg/dL
Creatinine	0.9 mg/dL	0.6–1.3 mg/dL
Calcium	9.2 mg/dL	8.5–10.5 mg/dL
Total protein	7.0 g/dL	6.0–8.3 g/dL
Albumin	4.2 g/dL	3.5–5.0 g/dL
Total bilirubin	0.8 mg/dL	0.1–1.2 mg/dL
Amylase	534 U/L	40–140 U/L
Lipase	656 U/L	0–160 U/L
AST (SGOT)	25 U/L	10–40 U/L
ALT (SGPT)	28 U/L	7–56 U/L
Alkaline phosphatase (ALP)	90 U/L	44–147 U/L
Total cholesterol	170 mg/dL	< 200 mg/dL
Triglycerides	120 mg/dL	< 150 mg/dL
HDL cholesterol	55 mg/dL	M: > 40; F: > 50 mg/dL
LDL cholesterol	95 mg/dL	< 100 mg/dL
VLDL (calculated)	24 mg/dL	2–30 mg/dL
Blood ethanol	< 10 mg/dL	< 10 mg/dL
CRP	12 mg/dL	< 1 mg/dL

**TABLE 2:** Hepatitis panel.

Test	Result
HBsAg	Negative
Anti‐HBs	Positive
Anti‐HBc	Negative
Anti‐HAV IgM	Negative
Anti‐HCV	Negative

### 2.2 Differential Diagnosis

The initial differentials for acute abdominal pain included gallstone pancreatitis, alcohol‐induced pancreatitis, hypertriglyceridemia, hypercalcemia, viral hepatitis, peptic ulcer disease, and DIP.

- *Gallstones* were excluded on the basis of normal abdominal ultrasonography and absence of biliary obstruction (Figure 1).
- *Alcohol‐related etiology* was ruled out as the patient denied alcohol use and serum ethanol was < 10 mg/dL (Table 1).
- *Metabolic causes* such as hypertriglyceridemia and hypercalcemia were excluded as lipid panel and calcium levels were within reference ranges (Table 1).
- *Viral hepatitis* was ruled out with negative serologies (Table 2).
- *Other causes* such as trauma, surgery, or endoscopic procedures were not applicable.

Given the temporal association with ciprofloxacin use, exclusion of common causes, and supportive imaging and biochemical findings, ciprofloxacin‐induced acute pancreatitis was considered the most likely diagnosis.

### 2.3 Outcome and Follow‐Up

**FIGURE 3:**
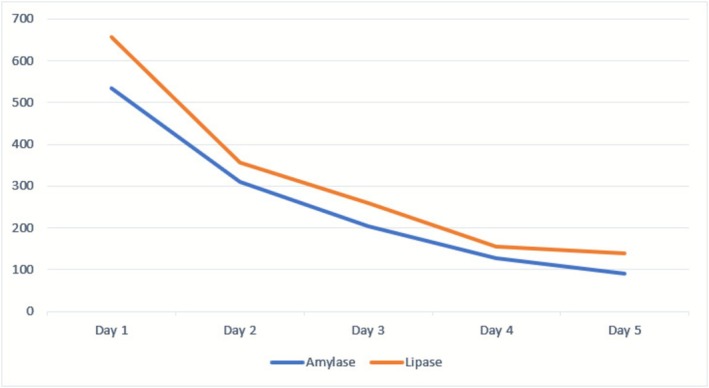
Amylase and lipase trend over days.

Ciprofloxacin was immediately discontinued. The patient was managed with aggressive intravenous fluid resuscitation, bowel rest (nil per os), opioid analgesia (tramadol), and supportive care. She demonstrated significant symptomatic relief, with progressive decline of serum amylase and lipase levels (Figure 3). By 96 h, pancreatic enzymes had normalized, and abdominal pain had resolved.

The patient was discharged on the fifth hospital day with complete clinical recovery. At follow‐up, she remained asymptomatic, with no recurrence of abdominal pain or biochemical abnormalities.

## 3 Discussion

According to the revised Atlanta classification, the diagnosis of acute pancreatitis requires the presence of at least two of the following three criteria: characteristic abdominal pain consistent with pancreatitis, serum amylase and/or lipase levels elevated to at least three times the upper limit of normal, and imaging findings (typically on CT, MRI, or ultrasound) consistent with acute pancreatitis [3]. In our case, the patient presented with acute‐onset epigastric pain and markedly elevated pancreatic enzymes, and contrast‐enhanced computed tomography (CECT) of the abdomen revealed definitive radiological evidence of pancreatitis. Based on the Atlanta criteria, the diagnosis of acute pancreatitis was confirmed.

The most common causes of acute pancreatitis include cholelithiasis (gallstones), alcohol use, hypertriglyceridemia, hypercalcemia, diabetes mellitus, and various hepatobiliary conditions such as viral hepatitis, cirrhosis, and cholangitis [1, 6, 7]. Extensive laboratory and imaging workup including abdominal ultrasound, MRCP, blood ethanol levels, lipid panel, and hepatitis panel were performed to rule out these common etiologies, all of which returned unremarkable.

After excluding more common causes and considering the onset of symptoms within 3 days of starting ciprofloxacin, a diagnosis of ciprofloxacin‐induced pancreatitis was made. DIP is considered a rare clinical entity, accounting for approximately 0.1%–2% of all acute pancreatitis cases [8]. The agents most frequently associated are antiretrovirals (e.g., didanosine), chemotherapeutics (e.g., asparaginase, cytarabine), antibiotics (e.g., tetracyclines, cotrimoxazole), steroids (e.g., dexamethasone, prednisolone, estradiol), 5‐aminosalicylates (e.g., sulphasalazine, mesalazine), antiepileptics (e.g., valproic acid, carbamazepine), antihypertensives (e.g., enalapril, lisinopril, losartan, furosemide), opiates (e.g., codeine), statins, and thiopurines (e.g., azathioprine, mercaptopurine) [2, 3, 8].

Among antibiotics, 5‐nitroimidazoles, tetracyclines, trimethoprim‐sulfamethoxazole, and isoniazid are the most frequently implicated agents in DIP. Fluoroquinolones, particularly ciprofloxacin, are less common but recognized causes [1]. Notably, ciprofloxacin's excellent pancreatic tissue penetration makes it valuable for preventing secondary infection in necrotizing pancreatitis, but its paradoxical potential to induce acute pancreatitis warrants clinician vigilance for this rare but serious adverse effect [9]. Although few case reports have documented ciprofloxacin‐associated pancreatitis, a multicenter observational study by Sung et al. (2007–2012) in patients receiving ciprofloxacin for infectious colitis suggested a possible association. In that study, ciprofloxacin‐induced pancreatitis occurred in approximately 3.1% of patients (7 out of 227), highlighting that this potential adverse effect should not be overlooked [4].

The onset of pancreatitis in those cases was relatively rapid, with a mean latency of 5.5 days (range: 4–7 days) following ciprofloxacin initiation. Notably, in our patient, symptoms developed within just 3 days, indicating an even shorter latency. While the exact mechanisms of ciprofloxacin‐induced pancreatic injury are not fully understood, such a short interval supports an idiosyncratic, delayed immunologic or T‐cell‐mediated response [10]. This may involve a hapten‐mediated mechanism, where a reactive metabolite of ciprofloxacin binds to host proteins, forming neoantigens that trigger an immune reaction. Alternatively, drug‐induced cellular stress may directly activate the immune system via co‐stimulatory signals to T lymphocytes. Emerging evidence also suggests that specific human leukocyte antigen (HLA) alleles may predispose individuals to such hypersensitivity reactions, further supporting an immune‐mediated etiology [1]. Additionally, a direct cytotoxic effect remains a possible contributing factor, as ciprofloxacin has been shown to induce cell cycle arrest and apoptosis in cultured human pancreatic cells [11].

To objectively assess causality, we applied the *Naranjo Adverse Drug Reaction Probability Scale*, a validated tool for evaluating the likelihood of an adverse event being drug‐related. The patient's score was 7, which corresponds to a *probable* adverse drug reaction (Table 3).

**TABLE 3:** Naranjo adverse drug reaction probability scale—assessment for ciprofloxacin‐induced pancreatitis.

Question	Yes (score)	No (score)	Do not know (score)	Case‐specific notes
1. Are there previous conclusive reports on this reaction?	Yes (+1)	No (0)	DK (0)	Published case reports and observational studies document ciprofloxacin‐induced pancreatitis.
2. Did the adverse event appear after the suspected drug was administered?	Yes (+2)	No (−1)	DK (0)	Symptoms began on day 3 of ciprofloxacin therapy.
3. Did the adverse reaction improve when the drug was discontinued or a specific antagonist given?	Yes (+1)	No (0)	DK (0)	Rapid symptomatic and biochemical improvement after ciprofloxacin withdrawal.
4. Did the adverse reaction reappear when the drug was readministered?	Yes (+2)	No (−1)	DK (0)	Not performed due to ethical concerns.
5. Are there alternative causes that could on their own have caused the reaction?	No (+2)	Yes (−1)	DK (0)	Common causes (gallstones, alcohol, hypertriglyceridemia, hypercalcemia, viral hepatitis) ruled out.
6. Did the reaction reappear when a placebo was given?	Yes (−1)	No (0)	DK (0)	Not applicable.
7. Was the drug detected in blood (or other fluids) in toxic concentrations?	Yes (+1)	No (0)	DK (0)	Not measured; no toxic level testing performed.
8. Was the reaction more severe when the dose was increased or less severe when the dose was decreased?	Yes (+1)	No (0)	DK (0)	Not applicable; fixed dose used.
9. Did the patient have a similar reaction to the same or similar drugs in any previous exposure?	Yes (+1)	No (0)	DK (0)	No prior fluoroquinolone exposure reported.
10. Was the adverse event confirmed by any objective evidence?	Yes (+1)	No (0)	DK (0)	Elevated amylase/lipase and CT scan consistent with acute pancreatitis.

*Note:* Total score: 7 → Probable ADR.

We also applied the *Badalov classification* for DIP, which stratifies drugs based on the quality of evidence, including case reports, rechallenge results, and latency patterns. Ciprofloxacin is classified as *Class I‐B*—drugs with at least one published case of positive rechallenge but without complete exclusion of other causes in all such reports (Table 4).

**TABLE 4:** Badalov classification for ciprofloxacin‐induced pancreatitis.

Class	Criteria	Ciprofloxacin placement	Justification for classification in this case
I	At least one case report with a positive rechallenge. Subclasses: I‐A (with exclusion of other causes) and I‐B (without exclusion).	I‐B	Published reports exist with positive rechallenge for fluoroquinolones (rare for ciprofloxacin); other causes excluded in our case but no rechallenge performed.
II	≥ 4 case reports with consistent latency period (≥ 75% cases).		Ciprofloxacin cases usually show 4–7 days latency; our patient's latency was 3 days.
III	≥ 2 case reports but without consistent latency pattern.		Not applicable here.
IV	Single case report without rechallenge or consistent latency.		Not applicable here.

DIP is generally considered mild to moderate in severity. The most crucial step in managing DIP is the immediate cessation of the suspected medication. Management is largely supportive and mirrors that of other etiologies. Patients are initially kept nil per os (NPO) to allow for bowel rest [6]. Intravenous fluid resuscitation is administered to maintain adequate hydration, alongside appropriate analgesia for pain control and antiemetics to manage nausea and vomiting. Proton pump inhibitors (PPIs) may also be used to reduce gastric acid secretion. Early refeeding is encouraged as soon as the patient is clinically stable and able to tolerate oral intake [2].

In some instances, specific pancreatic enzyme inhibitors can be used in the acute phase (like gabexate mesilate and camostat mesilate) to manage inflammation and high enzyme levels [4]. Pancreatic enzyme replacement therapy is used later or long‐term to address the deficiency of digestive enzymes that can result from moderate to severe pancreatitis, or when malabsorption symptoms such as steatorrhoea are present, regardless of the initial cause [2].

In previously reported cases, the prognosis of ciprofloxacin‐induced pancreatitis was favorable, with complete resolution following drug discontinuation. The average recovery time was 11.3 days (range: 8–15 days) [4]. In our case, the patient showed marked clinical improvement within just 4 days of ciprofloxacin withdrawal, as evidenced by normalization of pancreatic enzyme levels and resolution of abdominal pain. Notably, some reports have described even more rapid recovery within 40 h following levofloxacin cessation in a similar case of fluoroquinolone‐induced pancreatitis [6]. These findings underscore the variability in symptom onset and recovery time, reflecting individual susceptibility and reinforcing the need for heightened clinical awareness when diagnosing and managing suspected DIP.

A rechallenge test, which involves reintroducing the suspected agent to confirm causality through recurrence of pancreatitis, is considered the definitive method for establishing causality. However, due to the ethical concerns of provoking a potentially life‐threatening condition, rechallenge is generally avoided in clinical practice [2]. In our case, a rechallenge was not performed.

## 4 Conclusion

Ciprofloxacin‐induced acute pancreatitis is exceptionally rare but can occur within just a few days of therapy. In any patient who develops new or worsening epigastric pain during or shortly after fluoroquinolone use, clinicians should promptly measure pancreatic enzymes and consider cross‐sectional imaging. Immediate discontinuation of the offending agent, combined with aggressive supportive care (fluid resuscitation, bowel rest, and pain control), leads to rapid symptomatic and biochemical recovery in most cases. Maintaining a high index of suspicion for DIP ensures timely diagnosis and helps avoid potentially serious complications.

## Author Contributions

**Muskan Shrestha:** conceptualization, project administration, resources, writing – original draft, writing – review and editing. **Pratick Shrestha:** conceptualization, project administration, resources, writing – original draft, writing – review and editing. **Adarsha Mahaseth:** project administration, resources, writing – original draft, writing – review and editing. **Abhishek Shrestha:** resources, writing – review and editing. **Prabin Duwadee:** resources, writing – review and editing. **Rajat K. Shah:** resources, writing – review and editing.

## Funding

The authors have nothing to report.

## Consent

Written informed consent was obtained from the patient for publication of this case report and accompanying images. A copy of the consent form is retained by the authors and is available for review upon request.

## Conflicts of Interest

The authors declare no conflicts of interest.

## Data Availability

Data sharing is not applicable to this article as no datasets were generated or analyzed during the current study.
